# A New Migration Map of HIV-1 CRF07_BC in China: Analysis of Sequences from 12 Provinces over a Decade

**DOI:** 10.1371/journal.pone.0052373

**Published:** 2012-12-26

**Authors:** Zhefeng Meng, Ruolei Xin, Ping Zhong, Chiyu Zhang, Yassir F. Abubakar, Jingyun Li, Wei Liu, Xiaoyan Zhang, Jianqing Xu

**Affiliations:** 1 Shanghai Public Health Clinical Center and Institutes of Biomedical Sciences, Key Laboratory of Medical Molecular Virology of Ministry of Education/Health at Shanghai Medical College, Fudan University, Shanghai, China; 2 Department of AIDS/STD, Beijing Centers for Disease Control and Prevention, Beijing, China; 3 Department of AIDS/STD, Shanghai Municipal Centers for Disease Control and Prevention, Shanghai, China; 4 Pathogen Diagnostic Center, Institut Pasteur of Shanghai, Shanghai Institutes for Biological Sciences, Chinese Academy of Sciences, Shanghai, China; 5 Institute of Microbiology Epidemiology, Academy of Military Medical Science, Beijing, China; 6 Guangxi Provincial Center for Disease Control and Prevention, Nanning, China; 7 State Key Laboratory of Infectious Disease Prevention and Control, Beijing, China; Johns Hopkins School of Public Health, United States of America

## Abstract

**Background:**

As one of prevalence HIV-1 strains in IDUs in Asia, the origination and full transmission map of CRF07_BC is of great interested and remains unclear. In the study, we collected 769 CRF07_BC derived sequences (including 45 sequences generated in our laboratory) from 12 provinces in China for reconstructing transmission map. Meanwhile, ample historic epidemic evidences were also reviewed to assist sequences analysis.

**Methodology/Principal Findings:**

In the study, we collected 769 CRF07_BC derived sequences and identified 138 independent sequences from 12 provinces in China for subsequent phylogeographic tree analysis, Bayes Factor test and the estimation of state tMRCA. The analyses demonstrated that CRF07_BC was originated in 1993 in IDU in Yunnan province and then initially spread to Guangxi (eastern neighbor to Yunnan) in 1994, to Xinjiang (northwest) in 1995 and to Sichuan (northern neighbor to Yunnan) in 1996. The subsequent transmissions occurred from Yunnan to Liaoning (northeast) in 1997 and to Jiangsu in 1998. Interestingly, after the early introduction of CRF07_BC into Guangxi, Xinjiang and Sichuan, these three regions served as secondary epicenters for further spreading into Gansu, Ningxia, Qinghai, Beijing and Hunan during 1999–2001. These analyzed results are in accordance with early epidemic investigations.

**Conclusions/Significance:**

Our data not just reconstructed the migration map of CRF07_BC, but also firstly revealed the active roles of these secondary epicenters in the dynamic migration of CRF07_BC in China.

## Introduction

Human immunodeficiency virus type 1 (HIV-1) circulating recombinant forms (CRFs) 07_BC (CRF07_BC), descended from subtypes B′ and C, represents one of the most prevalent HIV-1 strains in Asia and has been devastating in IDUs for more than a decade in China [1]–[6]. Though the earliest CRF07_BC infections were reported in 1997 in Xinjiang [1]–[4], it had been believed that CRF07_BC was originated from Yunnan province [7]–[9]. However, this concept was challenged by a recent report proposed that Xinjiang might serve as an independent origin for CRF07_BC [10]. The limited published CRF07_BC sequences with confused epidemic and geographical information may have restrained to draw a definite conclusion. In addition, although CRF07_BC has spread across the majority of China, the currently published sequences covered only a few provinces, which render it a huge challenge to assemble spreading chains for CFR07_BC under this circumstance. Several important scientific questions remain elusive, including what evidences support Yunnan province as the origin site and epicenter of CRF07_BC, how did the transmission occur from the origin site to other regions, whether does unidentified and independent origin exist for CRF07_BC. To address those questions, more historic CRF07_BC sequences from early epidemic time and from different regions are required for analysis.

In present study, we generated 45 *gag* sequences in our own laboratories and merged them into sequence pools that contain all published sequences from both literatures and database. Our sequence pools cover 12 provinces that include almost all severely affected regions by CRF07_BC in China. Meanwhile, in order to corroborate the results from our sequence analyses, we traced back and reviewed all published literatures both in English and in Chinese on the earliest HIV-1 epidemic history in China[11]–[29]; Bayesian phylogeography method, a recently developed probabilistic method with more powerful capability to describe the most plausible scenario of geographic migration than previous methods [30], was employed to revisit the origin and phylogeography of HIV-1 CRF07_BC. Our results revealed that CRF07_BC was originated in 1993 in Yunnan province before spreading to Guangxi (eastern neighbor to Yunnan) in 1994, to Xinjiang (northwest) in 1995 and to Sichuan in 1996 (northern neighbor to Yunnan), both the origin site and secondary epicenters with early introduction of CRF07_BC played important roles in the dynamic migration of CRF07_BC in China, and both drug traffic and population migration may have significantly contributed to the complicated transmission pattern of CRF07_BC.

## Materials and Methods

### Patients and Sequences

45 plasma samples from 6 provinces (14 from Sichuan, 9 from Xinjiang, 7 from Guangxi, 8 from Beijing, 3 from Hunan and 4 from Jiangsu) were collected during 2004–2009 and used for amplifying *gag* gene. Written informed consents were obtained from all participants. The overall study was reviewed and approved by the Ethics Committee at SHAPHC (Shanghai Public Health Clinical Center). HIV-1 full-length or partial *gag* gene sequence was amplified as previously described [31]–[32]. By using an ABI 3730 Genetic Analyzer (Applied Biosystems), 30 full-length and 15 partial *gag* sequences (3 from Hunan, 4 from Jiangsu and 8 from Beijing) were generated successfully. The accession number of the new generated nucleotide sequences is deposited in the Genbank (JX392332–JX392376).

A total of 724 nucleotide sequences labeled as CRF07_BC, including 323 near full-length *gag* sequences, 361 *gp120* sequences and 40 C2V3 sequences, were retrieved from Los Alamos HIV Sequence Database (www.hiv.lanl.gov) and the China HIV/AIDS research database (http://hivdb.cn/content/hiv-db/china-db). After excluding all redundant sequences in HIV databases by the removal of sequences from the identical patient either as different clones or as different sampling date (refer to different months in a year), a total of 138 sequences (including the 45 newly generated *gag* sequences in our laboratory) were used for Bayesian phylogeography analysis (Table 1).

**Table 1:** The geographic distribution, sampling year and risk factor of 138 HIV-1 CRF07_BC sequences used in the study.

Geographic source	Sampling year	Risk factor	Gene regions		
			Full-length *gag*	Partial *gag*	*env* C2V3
Yunnan	1996–2002	IDUs	10	10	3
Guangxi	1998–2009	IDUs	8	8	5
Xinjiang	1997–2008	IDUs	22	22	14
Sichuan	1998–2007	IDUs	14	14	9
Liaoning	2000–2008	IDUs	10	10	
Jiangsu	2005	IDUs		4	
Gansu	2002	IDUs			1
Qinghai	2005	IDUs			2
Ningxia	2002–2005	IDUs			4
Beijing	2007–2009	IDUs, MSM		8	5
Hunnan	2005	IDUs		3	
Taiwan	2004–2005	IDUs			16
China subtotal			64	79	59

### Phylogenies and Temporal Dynamic Analyses

The phylogeographic and evolutionary analyses of CRF07_BC were performed on 64 full-length *gag* sequences, 79 partial *gag* sequences (HXB2∶892–1488 nt) (64 sequences matching with this fragment from 64 full-length *gag* sequences were also included), and C2V3 region in *env* sequences (HXB2∶7077–7391 nt). The datasets were edited and prepared with Bio-Edit V7.8 and Mega 5.2, and subsequently used for Bayesian MCMC evolutionary analyses. Before performing the Bayes MCMC analyses, the best nucleotide substitution model for all datasets was evaluated by the Mega 5.2. The Hasegawa-Kishino-Yano (HKY) nucleotide substitution model [33] with a gamma-distributed model among site rate variation using four rate categories (C4) [34], and a constant population size model were chosen as the best model for the Bayesian coalescence analyses [35]. Each MCMC analysis was run for at least 50 million generations and sampled every 10,000 generations in the BEAST V1.6.2 package. For constructing Maximum clade credibility (MCC) trees, the initial 25% of generated trees were discarded as burn-in and the leaving 3751 trees per run were summarized by using TreeAnnotator implemented in the BEAST V1.6.2 package. All those trees were examined and edited by using FigTree V1.3.1 (tree.bio.ed.ac.uk/software/figtree/), which was also used to estimate the evolutionary rates and the dates to tMRCA of various nodes on the MCC tree [36]. Posterior probabilities for the internal nodes were calculated from the posterior density of trees [37]. Statistical uncertainty in parameter estimates was reflected by the values of the 95% highest posterior density (HPD) credible region (CR). The posterior densities were calculated with 10% burn-in using Tracer V1.5.1. The program Tracer V1.5.1 (tree.bio.ed.ac.uk/software/tracer/) was used to check for the convergence and to determine whether effective sample size (ESS)>200. If the effective sample size is less than 200, the MCMC chain length would be elongated to 100 million.

### Phylogeographic Analysis and Bayes Factor Test

The BEAST V1.6.2 package could provide convenient phylogeographic analysis and Bayes factor test that determines the statistically significant phylogeographic links. Each sequence was first assigned a character state reflecting its sampling location. The Bayesian phylogeographic inference framework was performed to analyze the strength of the movement between geographic locations using a geographically explicit Bayesian MCMC method implemented in BEAST V1.6.2 package [30], [37]. This method can be used to infer the location state of the ancestral branch over the whole tree and to build a reversible diffusion rate matrix between previously defined locations accompanied with the evolutionary and coalescent parameters [30], [38]–[43]. Meanwhile, Bayes factor (BF) test was performed using the RateIndicatorBF tool, which was now incorporated into the BEAST V1.6.2 package [30]. If BF>3, the phylogeographic linkage between two locations was considered to be statistically significant [30]. In addition, the posterior probabilities for the ancestral geographic states were also calculated from the posterior density of trees summarized by using TreeAnnotator implemented in the BEAST V1.6.2 package [37].

### Visualization in the Google Earth

For Google Earth visualization, we have to associate each rate with two particular locations and their latitudes and longitudes. We use the same tab-delimited file prepared in Tree summary (http://beast.bio.ed.ac.uk/Tree_summary). The results obtained by the analysis above, including the TreeAnnotator outputted to the tree file and the rate indicators outputted to the discrete_rateMatrix.log file, was outputted to KML file, which can be opened and visualized in Google Earth. Moreover, the Bayes Factors were also produced by the rate indicators, which are frequently invoked to explain the diffusion process.

## Results

### The Origination of CRF07_BC in China

**Figure 1:**
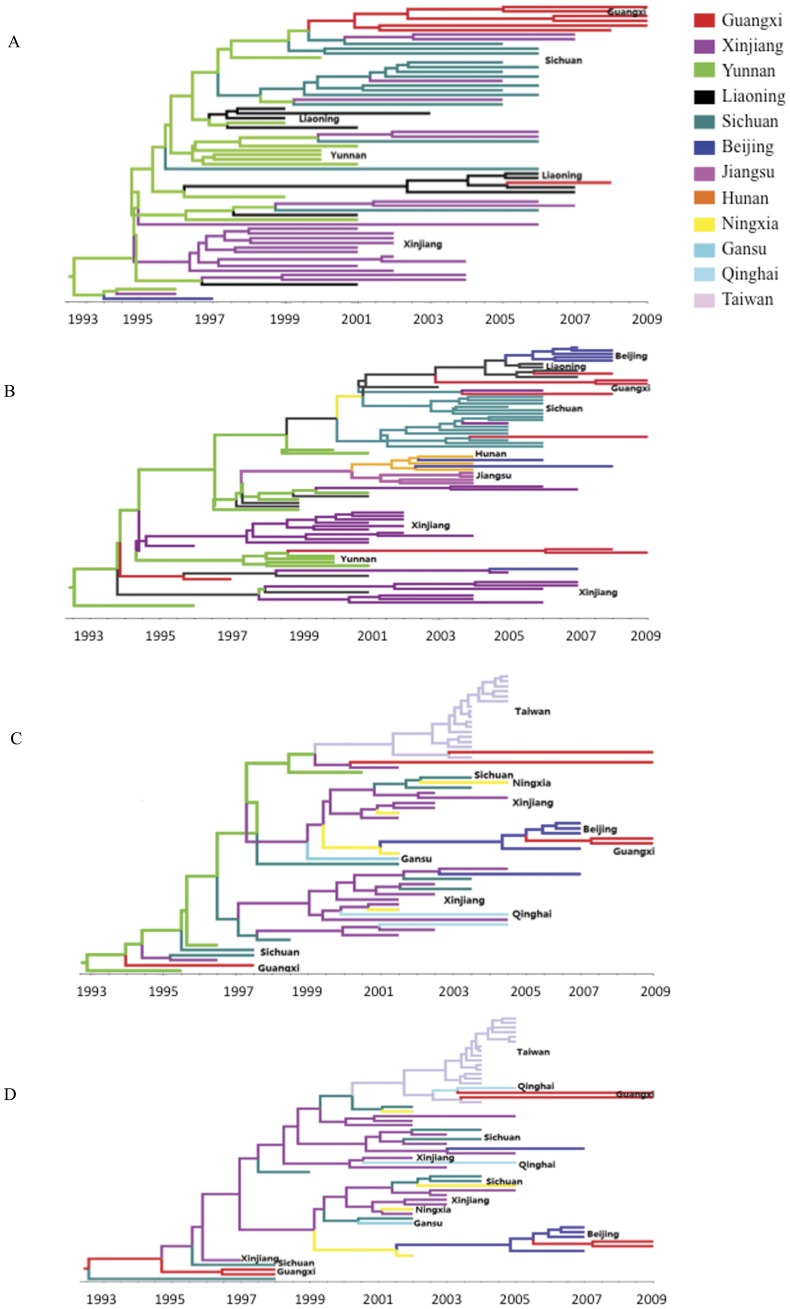
Maximum clade credibility trees of the HIV-1 CRF07_BC sequences. Ancestral geographic states were constructed using Bayesian phylogeographic inference framework implemented in the BEAST V1.6.2 package. The tree branches are colored according to their respective geographical regions. The root on the trees represents the most recent common ancestor (MRCA) of CRF07_BC. (A) The MCC tree constructed with the full-length *gag* sequences from 5 provinces; (B) The MCC tree constructed with the partial *gag* gene sequences (HXB2 2102–2601 nt) from 8 provinces; (C) The MCC tree constructed with the *env* C2V3 fragment (HXB2 7095–7328 nt) of subtype C from 9 provinces; (D) The MCC tree constructed on the *env* C2V3 fragment of subtype C from 8 provinces after the removal of sequences from Yunnan province.

**Figure 2:**
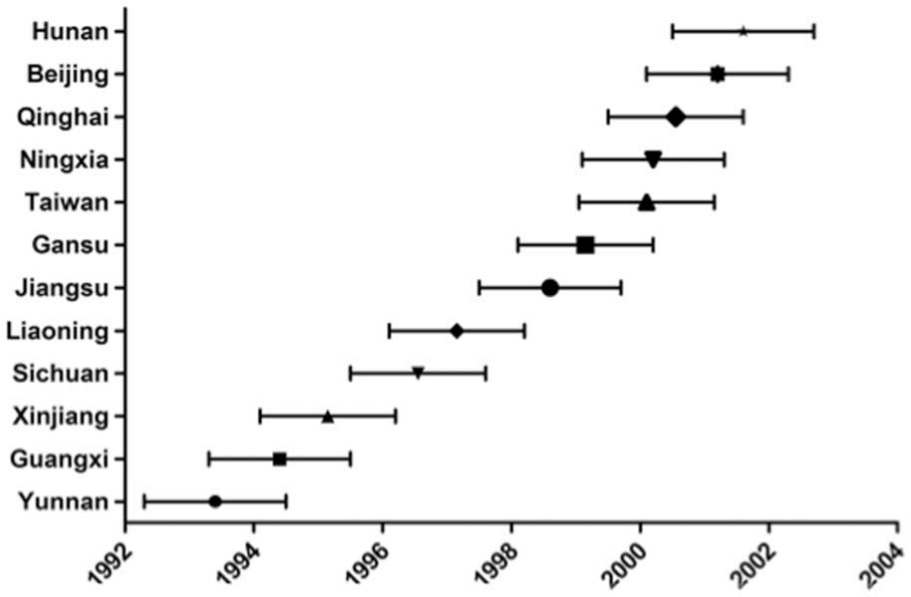
Estimated timescale of HIV-1 CRF07_BC from 12 provinces in China, the 95% highest posterior density (HPD) credible regions are provided as the range of state tMRCA.

**Figure 3:**
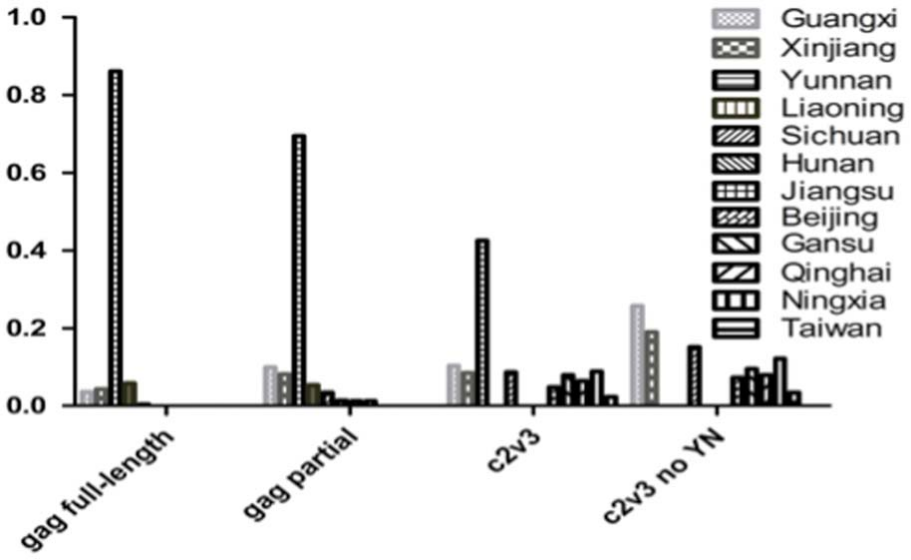
The root state posterior probabilities of CRF07_BC based on multiple genomic regions. The probabilities for the origin of CRF07_BC are shown in Y-axis, the designation of genomic fragments and the sequence-derived provinces are shown in X-axis.

The full-length *gag* dataset, including 64 sequences from 5 provinces (Yunnan, Guangxi, Xinjiang, Sichuan and Liaoning), was derived from samples collected during 1997–2008 and used to estimate the tMRCA of CRF07_BC (Fig. 1A). The rate of evolution of CRF07 _BC full-length *gag* gene was calculated by using Bayesian analysis under the HKY+γ substitution and constant size model, and was estimated to be (3.32–6.46)×10^−3^ substitutions/site/year. As summarized in Figure 2, the tMRCAs of CRF07_BC in China were dated to 1993 (95% credible region: 1992.3–1994.5), which is in agreement with previously published results [7]–[10]. It also indicated that CRF07_BC was originated in Yunnan with the highest root state posterior probabilities (0.86, Figure 3).

Phylogeographical tree of the full-length *gag* sequences was composed of regional clusters, including Guangxi cluster, Xinjiang cluster, Yunnan cluster, Liaoning cluster and Sichuan cluster. Notably, the isolates from Yunnan entered into each regional cluster and were supported as the ancestors for those regional clusters with high probabilities more than 0.9 (Fig. 1A, node probability not shown). In contrast, the isolates of Xinjiang entering into other regional clusters were not supported as the ancestor. These data supported that CRF07_BC was originated in Yunnan where was demonstrated as the earliest epidemic region by epidemiological investigations [7]–[29]. Even in the absence of Yunnan sequences, Xinjiang or Guangxi strains was also not supported as the definite root strain (data not shown). A similar conclusion was also drawn by analyzing 79 partial *gag* sequences that covered 8 provinces (Yunnan, Guangxi, Xinjiang, Sichuan, Beijing, Hunan, Jiangsu, Liaoning) (Fig. 1B). The tMRCA of HIV-1 CRF07_BC was estimated in 1993 (95% credible region: 1992.8–1994.9) and Yunnan strains were supported as the most possible root strains with the highest posterior probabilities (Fig. 3).

Based on the hypervariable region-stripped *env* region (Fig. 1C), the rate of evolution of C2V3 fragments was estimated to be (6.2–12.2)×10^−3^ substitutions/site/year, and the tMRCA of HIV-1 CRF07_BC corroborated the estimation with *gag* sequences and also dated to 1993 (95% credible region: 1991.2–1995.4) (Fig. 1c) [7], [8], [10]. Different from *gag* MCMC trees featured with regional clusters (Fig. 1a–b), the early *env* C2V3 from different regions formed two major clusters, one is clustered by sequences from Xinjiang, Sichuan, Ningxia, and the other is formed by strains from Xinjiang, Sichuan, Qinghai, indicating those early strains were genetically homologous and shared common ancestor. In addition, Taiwan CRF07_BC strains showed highly homologous and their common ancestor could be traced back to Yunnan strain, though the detailed routine of transmission from Yunnan to Taiwan remains unclear. When Yunnan strains were removed from the tree, the propabilities as an ancestor for strains from Guangxi, Xinjiang, Sichuan or other regions were all less than 0.3 (Fig. 1D and Fig. 3), which further suggested that Yunnan rather than other regions was the origin site. Taking together, it is likely that CRF07_BC was originated in Yunnan in 1993 and subsequently spread into other regions.

### Estimated Timeline for CRF07_BC Migration in China

The Phylogeographical trees constructed by full-length and partial *gag* and C2V3 sequences were used to estimate timeline of tMRCA for 12 Chinese provinces for CRF07_BC transmission in China. As shown in details in Figure 1 and summarized in Figure 2, Guangxi, an eastern neighbor province to Yunnan, is the earliest region for CRF07_BC entry and the transmission probably occurred in 1994.4 (95% CR: 1992.3–1996.5); The tMRCA for Xinjiang was dated to 1995.1 (95% CR: 1993.2–1997.3), to 1996.6 (95% CR: 1995.5–1997.6) for Sichuan which is neighbored to Yunnan in the north, to 1997.1 (95% CR: 1994.12–1999.3) for Liaoning (a province in Northern China with low HIV/AIDS prevalence) and to Jiangsu (located in Southeastern China) in 1998.6 (95% CR: 1996.5–2000.7). The tMRCAs of Gansu, Ningxia and Qinghai, which are all neighbored to Xinjiang, were dated to 1999.1 (95% CR: 1998.1–2001.2), 2000.2 (95% CR: 1999.1–2001.3) and 2000.5 (95% CR: 1999.5–2001.6), respectively. Further transmission occurred in Beijing (the capital city in northern China) in 2001.2 (95% CR: 2000.1–2002.3) and in Hunan (a province in central China) in 2001.6 (95% CR: 2000.5–2002.7) for. Finally, consistent with previously reports [7]–[10], the tMRCA of Taiwan was estimated to be 2000.1 (95% CR: 1999.1–2001.2).

Notably, due to the availability of earlier *env* C2V3 sequences than *gag* sequences in Sichuan, the estimated Sichuan tMRCA by *env* C2V3 is earlier than that by *gag* (1996.6, 95% CR: 1994.4–1998.7 vs 1997.5, 95% CR: 1995.4–1997.6), which suggested that Sichuan should be considered as the early epidemic region. In fact, Sichuan is neighbored to Yunnan in the north and the first CRF07_BC infections in Sichuan and Yunnan were almost simultaneously identified [16], [17], which implicated that the estimated tMRCA by *env* C2V3 is more reliable than that by *gag*.

### The Transmission Linkages Supported by Bayes Factor Test

Bayes factor was employed to evaluate the significance of phylogeographic linkages among different regions. BF test on three datasets not only supported Yunnan as the origin site and primary epicenter, but also suggested the existence of multiple transmission routes and secondary epicenters during CRF07_BC transmission in China. For instance, *gag* gene (full-length and partial gene) analyses indicated the transmission linkages among 8 provinces (Table 2), the strains from Yunnan had statistically significant geographic transmission linkages (BF>3) to strains from Xinjiang, Guangxi, Hunan, Jiangsu and Taiwan, and served as the primary epicenter; Meanwhile, Jiangsu, Xinjiang, Guangxi and Liaoning also showed statistically significant geographic transmission linkages (BF>3) with several other regions and acted as the secondary epicenters.

**Table 2:** The Bayes factors between defined locations of the three CRF07_BC gene regions sequences.

Origin	YN	GX	XJ	SC	LN	JS	NX	GS	QH	BJ	HN	TW
Yunnan (YN)		**14.21^a^**	**104.3^a^**	<3^a^	**21.7^a^**							
		**3.02^b^**	**42.28^b^**	<3^b^	**7.17^b^**	**4.09^b^**	<3^c^	<3^c^	**8.0^c^**	<3^b^	**3.39^b^**	**4.76^c^**
		**3.00^c^**	**11.33^c^**	**3.0^c^**						<3^c^		
Guangxi (GX)	<3^a^		**7.92^a^**	<3^a^	**6.32^a^**							
	<3^b^		<3^b^	<3^b^	**7.35^b^**	<3^b^						
	**3.0^c^**		<3^c^	<3^c^			<3^c^	<3^c^	<3^c^	**3.5^c^**	<3^c^	<3^c^
Xinjian (XJ)	<3^a^	<3^a^		**23.77^a^**	**3.63^a^**							
	**5.14^b^**	<3^b^		<3^b^	<3^b^	<3^b^				<3^b^	**19.18^b^**	
	**3.68^c^**	<3^c^		<3^c^			**11.66^c^**	<3^c^	<3^c^	<3^c^		**4.65^c^**
Sichuan (SC)	<3^a^	<3^a^	<3^a^		<3^a^							
	<3^b^	<3^b^	<3^b^		<3^b^	<3^b^					<3^b^	
	<3^c^	<3^c^	<3^c^				**6.41^c^**	<3^c^	<3^c^		**16.36^c^**	**5.48^c^**
Liaonin (LN)	<3^a^	<3^a^	<3^a^	**4.30^a^**								
	<3^b^	**26.07^b^**	<3^b^	<3^b^		<3^b^				**4.61^b^**	**11.96^b^**	
Jiangsu (JS)	<3^b^	<3^b^	<3^b^	**4.13^b^**	**3.13^b^**					**276.3^b^**	<3^b^	
Ningxia (NX)	<3^c^	<3^c^	<3^c^	<3^c^				<3^c^	<3^c^	<3^c^		<3^c^
Qinghai (QH)	<3^c^	<3^c^	**4.4^c^**	<3^c^			**3.00^c^**	<3^c^		<3^c^		<3^c^
Gansu (GS)	<3^c^	<3^c^	<3^c^	<3^c^			<3^c^		**3.19^c^**	<3^c^		<3^c^
Beijing (BJ)	<3^b^	**8.70^b^**	<3^b^	**3.65^b^**	<3^b^	<3^b^	<3^c^	<3^c^	<3^c^		<3^b^	<3^c^
	<3^c^	<3^c^	<3^c^	<3^c^	<3^c^	<3^c^					<3^c^	
Hunan (HN)	<3^b^	<3^b^	**44.16^b^**	<3^b^	<3^b^	<3^b^				<3^b^		
Taiwan (TW)	<3^c^	<3^c^	<3^c^	<3^c^	<3^c^	<3^c^	<3^c^	<3^c^	<3^c^	<3^c^	<3^c^	

Bayes factors above 3 (bold number) that represent statistically significant phylogeographic links between defined locations are shown.

a: full-length *gag*; b: partial *gag*; c: *env* C2V3.

BF test on *env* C2V3 dataset further supported Yunnan as the primary epicenter of CRF07_BC transmission. As shown in Table 2, Yunnan had significant transmission linkages with 5 regions, while Sichuan and Xinjiang had significant transmission linkages with 3 regions. Interestingly, when Yunnan sequences were removed from *env* C2V3 dataset, BF test showed that Sichuan and Xinjiang were the most important epicenters, which suggested the important role of Xinjiang and Sichuan during CRF07_BC transmission (data not shown).

### Probable Transmission Routes

**Figure 4:**
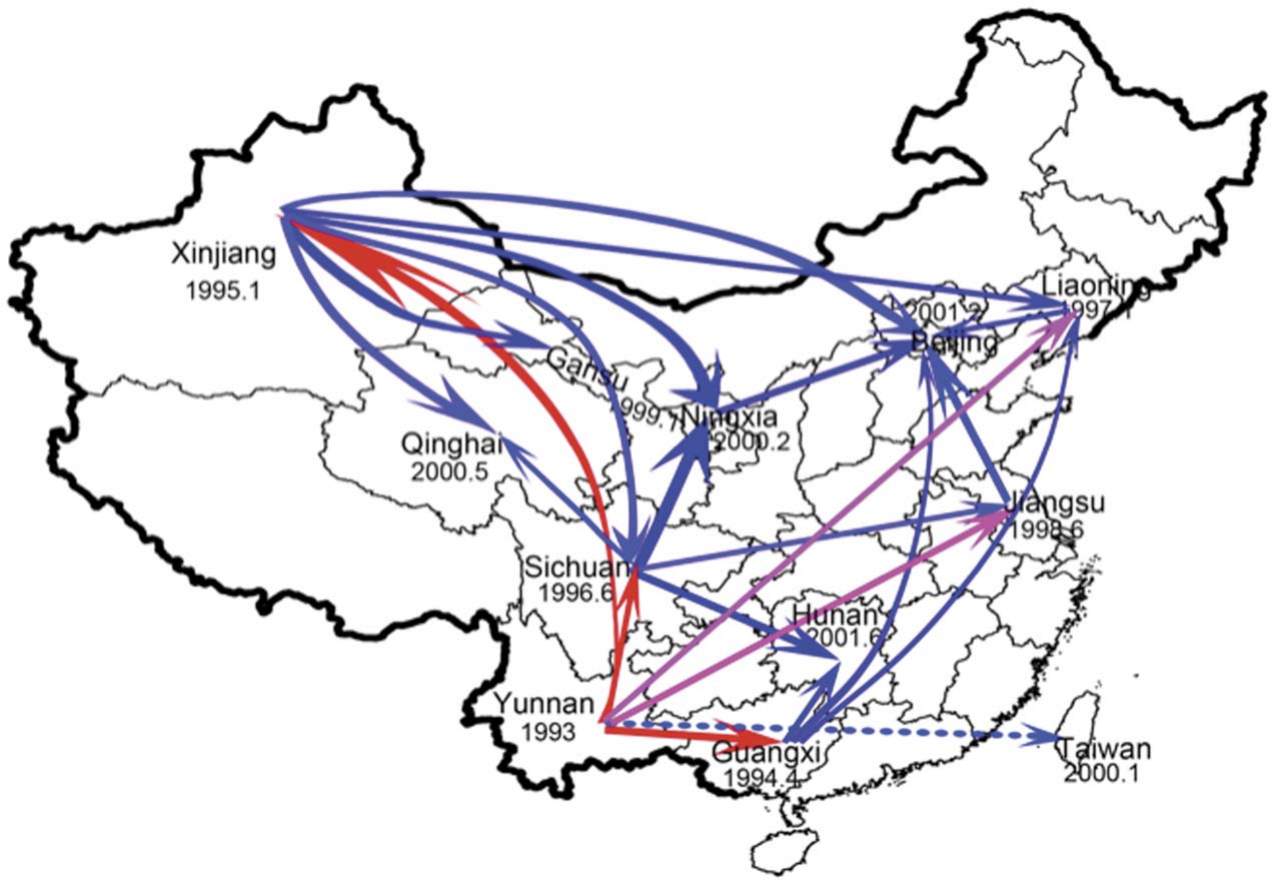
The probable transmission routes of HIV-1 CRF07_BC in China. CRF07_BC was originated in Yunnan province and initially spread to Guangxi, Xinjiang and Sichuan during 1994–1996 (red arrow), and then spread to Liaoning and Jiansu during 1997–1998 (pink arrow). All regions with the early introduction of CRF07_BC played a secondary epicenter role for further spreading (blue arrow). The dates for the entry of CRF07_BC for different provinces were labeled below the name of corresponding regions. Dotted arrow indicated indirect transmission for CRF07_BC.

The generated KML files by the TreeAnnotator were visualized in Google Earth (data not shown), which rebuild dynamic spreading of CRF07_BC in China as Figure 4 shown. Consistent with the analysis above of phylogeographical tree and Bayes factor test on three CRF07_BC sequence datasets, Yunnan was the most probable geographic origin for CRF07_BC strains (Fig. 4). The initial stage of spreading occurred in Guangxi (1994.4), Xinjiang (1995.1), and Sichuan (1996.5) after the generation of CRF07_BC in Yunnan, as red arrow pointed into these three regions in Figure 4. Notably, these earliest epidemic regions played critical roles in subsequent nationwide spreading of CRF07_BC, which were named as secondary epicenters during CRF07_BC transmission. Together with origin site, those secondary epicenters triggered further spread into Liaoning (1997.1), Jiangsu (1998.6), Gansu (1999.1), Taiwan (2000.1), Ningxia (2000.2), Qinghai (2000.5), Beijing (2001.2), Hunan (2001.6).

## Discussion

In the present study, we conducted an extensive phylogeny-based study on the date of origin and geographic spread pattern of CRF07_BC in China. It is for the first time that 769 sequences from 12 provinces were collected, and eventually 138 out of 769 were identified as independent and clean sequences and employed to build up the full epidemic and spread map for CRF07_BC. To choose the independent and clean sequences from HIV database for analysis, we removed all redundant sequences from either different sampling dates or different clones from the same patient. For instance, 298 near full-length *gag* sequences and 361 gp120 sequences submitted by Dr. Liu actually represented different clones from 9 patients [44], and only 9 *gag* and 9 *env* sequences were included in our analyses; 6 Xinjiang derived sequences included in the analyses by Dr. Takebe and Dr. Zhang were actually sampled from 4 patients [7]–[10], 4 of those 6 sequences submitted by our laboratory were derived from three patients [45], and the left two (97CN001 and CN54) were actually sampled from one identical patient [1]. Since those redundant sequences are highly homologous, it is rationalized that a bias may have been resulted from the inclusion of those redundant sequences for analysis. In addition, sequences with unidentified sampling dates or geography information were all excluded from our analyses, which were the cases for a number of sequences from Xinjiang and Yunnan.

Full length *gag* gene, partial *gag* gene (HXB: 2892-1488) and *env* hypervariable region C2V3 (HXB2∶7077–7391) were simultaneously included for MCMC analysis, all *gag* sequences cover the breakpoint and thereby contain both subtype B and subtype C fragments, *env* C2V3 belongs to subtype C. Consistent with the observation made by Dr. Zhang [10], MCMC analyses on those three datasets generated comparable results though a large fraction of *gag* and *env* C2V3 sequences were sampled from different regions at distinct prevalent period, and similar tMRCA and transmission linkages were observed among these common regions involved in these two gene datasets, such as for Yunnan, Xinjiang, Guangxi, Beijing. These results credited Bayesian MCMC as a reliable method for those analyses. It should be noticed that MCMC trees constructed by *gag* sequences were featured with regional clusters and each regional cluster was largely composed of corresponding region derived sequences, whereas the major clusters in *env* C2V3 constructed MCMC trees were formed by sequences from multiple regions. The reason for those observations is that *env* C2V3 sequences were sampled at relatively earlier period than *gag* sequences during CRF07_BC spreading, the close genetic relationship among different region derived strains in *env* C2V3 constructed MCMC trees indicated that those regional strains were derived from a consensus ancestor; However, after a prolonged circulation at a certain region, those early strains gradually gained their regional features and formed regional sub-cluster, which was manifested by *gag* constructed MCMC trees.

All our data pointed the origin of CRF07_BC toward Yunnan. The analyses with *gag* sequences showed very high probabilities of the root strain for Yunnan strains (≥0.85); Though only 3 Yunnan sequences are available in *env* C2V3 constructed MCMC trees, the probability for Yunnan strains as the root strain remained much higher than that for other strains, importantly, when Yunnan sequences were removed from the analysis, none regional strains was inferred as the definite root strain and the probability of root strain was shared by strains derived from Guangxi, Xinjiang and Sichuan, which indirectly supported Yunnan as the origin site for CRF07_BC.

Several lines of evidences proved that heroin smuggling, which is started at the Triangle area bordered to Yunnan, dictated the spreading of CRF07_BC [1]–[3], [11]–[29], since CRF07_BC was largely circulating in IDUs at the early spreading stage, and two heroin smuggling routes were considered to account for the spreading of CRF07_BC from Yunnan to Guangxi (eastern route) and from Yunnan to Xinjiang (northern route). For the latter route, more regions, including Sichuan, Ningxia, Qinhai and Gansu, were located between Yunnan and Xinjiang and thereby were likely to be involved at the early stage. Since neighbored to Yunnan in the north, Sichuan was speculated as one of the earliest region for the introduction of CRF07_BC and may have played an important role in the further transmission of CRF07_BC. Indeed, both social-demographic and early HIV-1 epidemic information supported the speculation above. First, as the major HIV-1 affected population, local Yi ethnic people in Yunnan routinely exchange and communicate with Yi people who are living in the bordered area to Yunnan in Sichuan, which undoubtedly facilitated the immediate transmission of CRF07_BC from Yunnan to Sichuan; Second, the CRF07_BC infection in Yunnan and Sichuan were simultaneously identified as the earliest Reports in China in 1998 [16], [17]. As a region not noticed by previous reports [7]–[10], Sichuan was for the first time supported as an important secondary epicenter for CRF07_BC spreading by MCMC tree and BF test in addition to previous epidemic evidences.

Similar to Sichuan, Guangxi was also not included in previous reports [7]–[10]. In fact, the first HIV infection in Guangxi was reported in 1989, as early as the report from Yunnan [18]–[21], the co-circulation of Thai-B and C subtypes in IDUs was reported as early as in 1995 in Guangxi by Chinese Journals [19]–[21], which was in agreement with the estimated Guangxi tMRCA, therefore, Guangxi was speculated as the earliest region for the migration of CRF07_BC. However, too few sequences from Guangxi are available in database, it is hard to draw a conclusion and thereby previous analyses may intently not include this region. In this study, we collected 7 full-length *gag* sequences from Guangxi for the analysis, which conferred the probability to precisely estimate the state tMRCA in Guangxi. Our analyses dated the introduction of CRF07_BC into Guangxi to 1994. These results were also in accordance with early HIV transmission history in this region [19]–[21].

The timeline of state tMRCA did not always match the distance from Yunnan to epidemic regions along the heroine smuggling route. For examples, the introduction of CRF07_BC into Xinjiang (the farthest northwestern region) occurred at very early phase, even earlier than the entry into those regions located between Yunnan and Xinjiang, including Sichuan, Ningxia, Qinhai and Gansu. Two reasons may be responsible for this issue, first, according to Berry’s theory [46], CRF07_BC, as the first HIV-1 strain entered into Xinjiang, initiated a rapid spreading among IDUs since no pre-existing immunity and no viral competition constrained this initial spreading, which built up a larger CRF07_BC infected population in a shorter time in Xinjiang than Sichuan where B subtype was the earlier circulating strain than CRF07_BC; Second, the appearance of heavily drug abuse in Xinjiang is earlier than most inner region along the heroine smuggling route(unpublished data), which subsequently facilitate to immigrant of IDUs in whole country and the crucial roles of Xinjiang during CRF07_BC spread.

Since Xinjiang strains played an important role during CRF07_BC spreading, Dr. Zhang rationalized that Xinjiang should be considered as an independent origin site in his analysis [10]. However, our analyses of all three datasets supported the early introduction of CRF07_BC into Xinjiang but not the origin site; Our results were further supported by epidemiological evidence and previous studies. Actually, Chinese national reports showed that no HIV infection was identified in Xinjiang until 1995 [12], [13], [15], which is in agreement with our estimated Xinjiang tMRCA but not with the previously estimated tMRCA of CRF07_BC. In addition, CRF07_BC is the only BC recombinant form in Xinjiang whereas multiple BC recombinants were identified in Yunnan and Guangxi [11]–[15], including CRF07_BC, CRF08_BC and many more BC recombinants which were generated through a similar mechanism and from a common source [3], [6], [7], [47]. The fact that CRF08_BC has been widely spread in Guangxi and Yunnan [48], [49] but not in Xinjiang [50], further supports our results that CRF07_BC more likely originated in Yunnan rather than Xinjiang.

In addition to Xinjiang, other regions with early introduction of CRF07_BC also played the role as secondary epicenters in the further spreading of CRF07_BC, including Guangxi, Liaoning and Sichuan. It should be noticed that the introductions of CRF07_BC into Jiangsu, Hunan and Beijing were hard to be explained by the main heroin smuggling routes but accommodated with the locally rapid expansion of IDU populations [22]–[24], [28] and their exchanging migration among different regions. The spreading pattern was further complexed by the engagement of MSM populations who are featured with frequent migration among cities [28]. Beijing as the capital city in China received immigrants from all over the country; therefore, multiple introductions of CRF07_BC from different regions were identified in our analyses. Taking together, the population migration instead of drug smuggling played more important role in the subsequent nationwide spreading of CRF07_BC, which was further enhanced under the restrict control of drug smuggling by Chinese government in recent years. Finally, CRF07_BC has disseminated beyond Mainland China and transmitted into Taiwan around 2000, which is in accordance with previously descriptions [5], [29]. It was believed that CRF07_BC was introduced into Taiwan probably via southeastern provinces in China (i.e. Fujian and Guangdong, which have strong social and demographic ties with Taiwan) [5], [29], which was also indirectly supported by our results by the indication of the significant linkage between CRF07_BC strains from Taiwan and Yunnan.

Altogether, our analyses provided more details for CRF07_BC migration in China and demonstrated that CRF07_BC was originated in Yunnan and initially spread to Guangxi, Xinjiang and Sichuan. Interestingly, those regions with the early introduction of CRF07_BC played a role as the secondary epicenters for further spreading. Xinjiang, due to its extensive migration of populations to other regions, played a crucial role in the subsequent spreading of CRF07_BC to other areas. A two-phase spreading of CRF07_BC was observed, the early spreading of CRF07_BC was likely to be triggered by drug smuggling and the subsequent spreading was probably more influenced by population migration.
